# Prevalence of Physical Activity and Sedentary Behavior Patterns in Generally Healthy European Adults Aged 70 Years and Older—Baseline Results From the DO-HEALTH Clinical Trial

**DOI:** 10.3389/fpubh.2022.810725

**Published:** 2022-04-14

**Authors:** Michèle Mattle, Ursina Meyer, Wei Lang, Noemi Mantegazza, Michael Gagesch, Richard Mansky, Reto W. Kressig, Andreas Egli, E. John Orav, Heike A. Bischoff-Ferrari

**Affiliations:** ^1^Center on Aging and Mobility, University Hospital Zurich, City Hospital Zurich - Waid, and University of Zurich, Zurich, Switzerland; ^2^Department of Aging Medicine and Aging Research, University Hospital Zurich and University of Zurich, Zurich, Switzerland; ^3^University Department of Geriatric Medicine FELIX PLATTER, Basel University, Basel, Switzerland; ^4^Department of Biostatistics, Harvard T. H. Chan School of Public Health, Boston, MA, United States; ^5^University Clinic for Aging Medicine, City Hospital Zurich - Waid, Zurich, Switzerland

**Keywords:** sedentary behavior, physical activity, older adults, lifestyle, prevalence, healthy aging, active aging

## Abstract

**Background:**

Physical activity (PA) is important for healthy aging and disease prevention whereas sedentary behavior (SB) accelerates health deterioration.

**Aim:**

To investigate activity profiles regarding PA and SB among generally healthy European older adults.

**Methods:**

Meeting PA recommendations was defined as ≥150 min/week of moderate and/or ≥75 min/week of vigorous PA. A cut-off of ≥5.5 h/day was used to define time spent with SB. We present prevalence of PA and SB overall and by sex, age, BMI, and country. We examined correlates with multivariate logistic regression models.

**Results:**

Two thousand one hundred and fifty-five DO-HEALTH participants completed baseline information on activity profiles [mean age 74.9 years (SD 4.5), 61.8% women]. Overall, 62.2% met PA recommendations and overall, 37.1% spent ≥5.5 h/day with SB. Younger participants (70–74 years), men, and those with BMI <25 kg/m^2^ met PA recommendations more often. Per country, prevalence of meeting PA recommendations were: Austria 74.4%, France 51.0%, Germany 65.6%, Portugal 46.5%, and Switzerland 66.7%. Regarding SB, prevalence did not differ in all subgroups. In multivariate logistic regression analyses, being male, younger age, lower MoCA scores, and higher SPPB score were associated with greater odds, whereas higher BMI, more years of education, higher GDS score, and residing in Portugal were associated with lower odds of meeting PA recommendations. High BMI and higher MoCA scores were associated with greater odds of high SB.

**Conclusion:**

Individualized public health efforts may be warranted even in active older adults, as profiles were less favorable in subgroups of older age, female sex and higher BMI.

## Introduction

In Europe, the share of the population being older than 65 years is expected to rise from 20% in 2020 up to 30% in 2050 (1). Age-related chronic diseases such as cardiovascular diseases, diabetes, cancer, and dementia represent a considerable burden to the affected individual, the society, the economy, and the health care system (2).

Physical activity (PA) plays a key role in the prevention of chronic diseases and reduces mortality (3). Likewise, engaging in PA reduces loss of autonomy by prevention of frailty (4), as well as through high effectiveness to prevent falls (5). Consequently, sufficient PA strongly influences an older person's trajectories of “active and healthy aging” (6). Currently, the World Health Organization (WHO) recommends ≥150 min of moderate or ≥75 min of vigorous PA per week for all adults, with a specification for older adults to engage in multicomponent PA of at least moderate intensity on 3 or more days a week (7). However, about 55–83% of women and 47–74% of men do not meet these recommendations (8).

Spending high amounts of time with sedentary behavior (SB) has been associated with aggravated decline of physical function (9), decreased muscle health (10), and subsequently increased risk of falling (11). Furthermore, a dose-response relationship between the amount of SB and mortality risk has been reported in community-dwelling older adults (12).

Higher PA levels and limited time spent with SB are proposed to be independently related to better health outcomes (13). As it is challenging to meet PA recommendations for many older adults, especially in presence of multimorbidity (14–16), replacing SB with light PA may be the stepping-stone toward eventually spending more time with moderate or vigorous PA (17).

Consequently, the WHO emphasizes the importance of decreasing SB in addition to meeting PA recommendations (7). Nonetheless, the Eurobarometer surveys show that overall, the total time spent with SB/day increased between 2002 and 2017 (18). Importantly, a review of large cohort studies found that older adults spent between 5 and 9 h/day with SB (19).

Aging research increasingly investigates conditions leading to increased SB and insufficient PA. Nevertheless, a consistent operational definition of a phenotype of older adults living active vs. inactive, and sedentary vs. non-sedentary lifestyles is still missing today. Further, as the definition of SB was only introduced in 2012, limited research is available about prevalence of SB among older adults, especially within subgroups of oldest age, sex, or geographical origin (19). To establish a risk profile in clinical care and foster suitable interventions, knowledge about living circumstances and behavioral patterns is essential.

The DO-HEALTH clinical trial offers a unique data set from extensively phenotyped community-dwelling generally healthy older adults aged 70+ from five European countries (20). The first aim of this secondary analysis of baseline data from DO-HEALTH is to describe the prevalence of PA, SB, and the combination of these two behavioral patterns in a generally healthy community-dwelling older adult population. Secondly, this study aims to characterize participants meeting PA recommendations and/or engaging in high amounts of SB regarding socio-demographic characteristics, as well as physical and cognitive function.

## Methods

### Study Design and Participants

The DO-HEALTH clinical trial randomized 2,157 community-dwelling healthy older adults aged 70 and older to vitamin D, omega-3 fatty acids, and simple home exercise program, according to the 2 × 2 × 2 factorial design. Participants were recruited at seven study centers in five European countries. The design variables used for randomization stratification in the DO-HEALTH trial were age, sex, experience of a fall in the year prior to study inclusion, and study site. Participants were recruited from the community trough mailing lists of, i.e., retirement authorities and community services, and trough advertisements in newspapers and other media. The study design and the main results have been published elsewhere (20, 21).

### Assessment of PA and SB

Participants reported the types and average time spent with PA and SB per week within the past year with an excerpt of the Nurses' Health Study questionnaire (NHS PAQ) (22). The NHS PAQ is a validated self-reporting questionnaire covering the time spent with different leisure-time PA, time spent standing or walking, time spent with SB, number of days exercised per week, number of stair flights climbed per week, and rating of usual gait speed outdoors (22). Participants filled out the NHS PAQ independently on a tablet. Answers given as intervals of time were coded as means of the intervals (Supplement 1).

We classified the intensity of activities reported with the NHS PAQ following the physical activities compendium using metabolic equivalents of tasks (METs) as light (<3 METs), moderate (3–6 METs) and vigorous (≥6 METs) (23). Then, we calculated the reported time per week spent with moderate and vigorous activities (23). To account for over-reporting of amounts of activities (outlier data), we capped the sum of moderate PA at 35 h/week and the sum of vigorous PA at 21 h/week. We defined participants meeting PA recommendations if they engaged in either ≥150 min/week of moderate, and/or ≥75 min/week of vigorous PA (Supplement 2) (7).

We calculated the reported hours/day of SB based on questions q4.3–q4.5 of the NHS PAQ (Supplement 1). There is no established definition of “high SB” or a cut-off available to account for time spent with SB considered as health threatening (19). In compliance with a special report by the Swiss Federal Office for Public Health (FOPH), we set the cut-off for the binary variable SB (0.1) at 5.5 h/day (24). We capped the sum of SB at 24 h/day.

### Assessment of Participant Characteristics

All DO-HEALTH study participants completed a comprehensive baseline assessment including questionnaires and standardized assessments of physical and cognitive function. Physical function was assessed with the Short Physical Performance Battery (SPPB) (25), and handgrip strength measured using a Martin Vigorimeter (26). Cognitive function was assessed with the Montreal Cognitive Assessment (MoCA) (27), and the Mini-Mental State Examination (MMSE) (28). The number of comorbidities was assessed with a self-administered questionnaire (Sangha's score) (29). Health-related quality of life was assessed with the EuroQol 5 Dimensions 3 Levels (EQ-5D-3L) and self-rated health was assessed by the EQ-5D-3L vertical visual analog scale (VAS) (30). Depression was assessed with the 15 items Geriatric Depression scale (GDS) (31).

### Statistical Analyses

Descriptive statistics are presented with frequency counts and percentages for categorical variables and mean ± standard deviation (SD) or median with interquartile range (IQR) for continuous variables depending on the normality of their distribution. In a first step, bivariate associations were examined using the Chi-square test between two categorical variables (pre-specified subgroup analyses regarding categories of age (70–74 years/≥75), sex (female/male), body mass index (BMI, ≥25/ <25), and country of residence (reference = Switzerland).

Secondly, dichotomous outcomes of meeting PA recommendations (yes/no, model 1) and spending ≥5.5 h/day with SB (yes/no, model 2) were analyzed using separate multivariable logistic regression models. The following variables simultaneously entered both models: age, sex, experiencing a fall prior to inclusion, country of residence, BMI, current smoking, living alone, years of education, being depressed (GDS), cognitive function (MoCA score), multimorbidity (≥2 comorbidities), polypharmacy (taking ≥5 medications), and physical function (Grip Strength and SPPB score).

Additional analyses were conducted by including SB (spending ≥5.5 h/day: yes/no) in the multivariable logistic regression model of the odds of meeting PA recommendations; Similarly, the covariate meeting PA recommendations (yes/no) was added in the multivariable logistic regression model of the odds of spending ≥5.5 h/day with SB.

All analyses were performed using SAS® software, Version 9.4 of the SAS System for Windows and RStudio Version 4.0.3. The significance level was fixed at 0.05.

## Results

### Baseline Characteristics of Study Population

We included 2,155 of all 2,157 DO-HEALTH participants with complete baseline NHS PAQ profiles. Mean age was 74.9 years (SD 4.5), and 61.8% were women (Table 1). As per design of the clinical trial, 41.9% of participants reported having experienced a fall 12 months prior to study inclusion. Overall, 5.8% reported current smoking. The mean number of comorbidities was 3.3 (SD 3.0) and the mean number of medications taken 3.2 (SD 2.8).

**Table 1:** Baseline characteristics of the study population, a) by meeting or not meeting PA recommendations, b) by reporting ≥5.5 hours/day of SB or not.

		**a)**		**b)**	
	**Overall**	**Meeting**	**Not meeting**	**≥5.5 hours/**	**<5.5 hours/**
		**PA recommendations**	**PA recommendations**	**day of SB**	**day of SB**
***n*** (%)^*^	2,155	1,341 (62.2)	814 (37.8)	800 (37.1)	1,355 (62.9)
**Age, (yrs)**					
Mean (SD)	74.9 (4.5)	74.3 (3.9)	76.0 (5.0)	75.0 (4.4)	74.9 (4.5)
70–74, *n* (%)	1,236 (57.4)	838 (67.8)	398 (32.2)	447 (36.2)	789 (63.8)
>75, *n* (%)	919 (42.6)	503 (54.7)	416 (45.3)	353 (38.4)	566 (61.6)
**Sex, *n* (%)**					
Female	1,331 (61.8)	751 (56.4)	580 (43.6)	473 (35.5)	858 (64.5)
Male	824 (38.2)	590 (71.6)	234 (28.4)	327 (39.7)	497 (60.3)
**Prior fall, *n* (%)**					
Yes	902 (41.9)	541 (60.0)	361 (40.0)	348 (38.6)	554 (61.4)
No	1,253 (58.1)	800 (63.8)	453 (36.2)	452 (36.1)	801 (63.9)
**BMI, (kg/m^2^)**					
Mean (SD)	26.3 (4.3); (*n* = 2,154)	25.8 (4.0)	27.2 (4.6)	26.6 (4.4)	26.2 (4.2)
≥25	1,286 (59.7)	746 (58.0)	540 (42.0)	498 (38.7)	788 (61.3)
<25	868 (40.3)	595 (68.5)	273 (31.5)	302 (34.8)	567 (65.2)
**Current smoking, *n* (%)**					
Yes	126 (5.8)	48 (38.1)	78 (61.9)	55 (43.7)	71 (56.3)
No	2,029 (94.2)	1,293 (63.7)	736 (36.3)	745 (36.7)	1,284 (63.3)
**Years of education**, mean (SD)	12.6 (4.3); (*n* = 2,153)	12.8 (4.1)	12.3 (4.6)	12.9 (4.4)	12.5 (4.3)
**MoCA score**, mean (SD)	25.7 (3.4); (*n* = 2,151)	25.7 (3.1)	25.5 (3.6)	26.0 (3.2)	25.5 (3.5)
**MMSE score**, mean (SD)	28.5 (1.5)	28.4 (1.6)	28.6 (1.5)	28.5 (1.5)	28.5 (1.5)
**Number of comorbidities**, mean (SD)	3.3 (3.0); (*n* = 2,154)	4.0 (3.2)	2.9 (2.9)	3.5 (3.2)	3.2 (2.9)
**Number of medications/polypharmacy**					
Mean (SD)	3.2 (2.8)	3.7 (3.0)	2.9 (2.6)	3.3 (2.9)	3.1 (2.7)
<5, *n* (%)	1,571 (72.9)	1,026 (65.3)	545 (34.7)	575 (36.6)	996 (63.4)
≥5, *n* (%)	584 (27.1)	315 (53.9)	269 (46.1)	225 (38.5)	359 (61.5)
**Health-related quality of life** (EQ-5D-3L) score, mean (SD)	0.901 (0.139); (*n* = 2,152)	0.918 (0.128)	0.873 (0.151)	0.891 (0.146)	0.907 (0.134)
**Self-rated health** (EQ-5D-3L VAS) score, mean (SD)	81.2 (14.9); (*n* = 2,152)	83.4 (13.7)	77.8 (16.2)	81.6 (14.8)	81.0 (15.0)
**Geriatric depression scale** (GDS) score, mean (SD)	1.8 (2.3); (*n* = 2,127)	1.4 (2.0)	2.4 (2.7)	1.8 (2.4)	1.7 (2.3)
**Living alone, *n* (%)**					
Yes	900 (41.8)	522 (58.0)	378 (42.0)	339 (37.7)	561 (62.3)
No	1,255 (58.2)	819 (65.3)	436 (34.7)	461 (36.7)	794 (63.3)
**Physical function**					
mean (SD)					
SPPB (score)	10.9 (1.5); (*n* = 2,151)	11.15 (1.2)	10.4 (1.8)	10.8 (1.5)	10.9 (1.5)
Grip Strength dominant hand (kPa)	60.2 (18.6); (*n* = 2,150)	62.6 (18.5)	56.2 (18.1)	61.3 (18.6)	59.5 (18.5)
**Country, *n* (%)**					
Austria	199 (9.2)	148 (74.4)	51 (25.6)	76 (38.2)	123 (61.8)
France	300 (13.9)	153 (51.0)	147 (49.0)	124 (41.3)	176 (58.7)
Germany	349 (16.2)	229 (65.6)	120 (34.4)	119 (34.1)	230 (65.9)
Portugal	301 (14.0)	140 (46.5)	161 (53.5)	101 (33.6)	200 (66.4)
Switzerland	1,006 (46.7)	671 (66.7)	335 (33.3)	380 (37.8)	626 (62.2)

* *DO-HEALTH included total 2,157 participants. Two participants had missing values for the NHS PAQ and therefore were excluded*.

*For the overall, % sum up to 100 in columns, within sections a) and b), % sum up to 100 in rows*.

*Prior fall: reporting of a fall in the 1-year period before study start. BMI: Body mass index, calculated as weight in kilograms divided by height in meters squared (kg/m^2^). BMI values ≥25 reflect overweight and values ≥30 obesity. MoCA, Montreal Cognitive Assessment: screening test for mild cognitive dysfunction with a range of 0 to 30 points, in which higher scores are better and scores >26 suggest normal cognitive function. MMSE: Mini-Mental State Examination, measures cognitive impairment with a range of 0 to 30 points, in which higher scores are better and scores >24 suggest normal cognitive function. MMSE of ≥24 was one of the inclusion criteria for the DO-HEALTH clinical trial. Comorbidities were assessed with a self-administered questionnaire to assess comorbidities (Sangha's Score). Health-related quality of life: assessed by the EuroQol 5 Dimensions 3 Levels (EQ-5D-3L). Scores range from <0 to a maximum of 1 point, in which 0 means a health state equivalent to death, negative values are equivalent to a health state worse than death, and 1 is equivalent to perfect health. Self-rated health: assessed by the EQ-5D-3L vertical visual analog scale (VAS), which ranges from 0 to 100 points, in which higher scores are better. GDS: Geriatric Depression scale, Questionnaire to assesses depression. Scores range from 0 to 15, 0–5 points are considered normal, 5–10 points translate to light to moderate depression, 11–15 to severe depression. SPPB: Short Physical Performance Battery, standardized assessment battery to test lower extremity function. Scores range from 0 to 12, in which higher scores are better. Grip Strength of the dominant hand was measured with a Martin Vigorimeter (in kilopascal, kPa)*.

Overall, participants reported a median of 18.5 (IQR: 9.5, 38.7) h/week spent with light PA, 2.6 (IQR: 0.7, 7.3) h/week spent with moderate PA, 0.2 (IQR: 0.5, 1.4) h/week spent with vigorous PA, and a median sum of 3.9 (IQR: 2.1, 6.7) h/day spent with SB (Supplement 3). Interestingly, participants reporting to spend ≥ 5.5 h/day with SB at the same time reported overall more time spent with PA.

Walking was the most common PA, followed by gymnastics (including Yoga, stretching, figure training) and “other activities (e.g., lawn mowing)”. Regarding SB, median reported time spent watching TV and median time “sitting at home” were both 1.1 h/day (IQR: 0.5, 2.2, for both).

### Prevalence of PA

**Figure 1:**
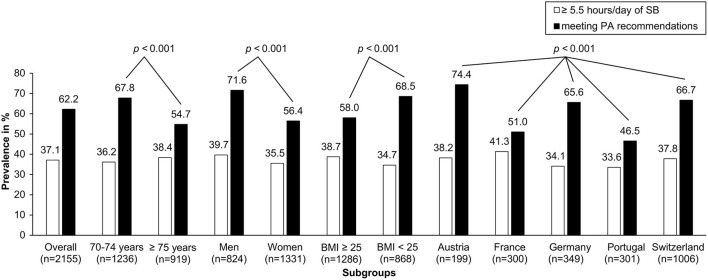
Prevalence of participants meeting PA recommendations and prevalence of participants spending ≥ 5.5 hours/day with SB; in %, per subgroup. For BMI, data of 2,154 participants was available. *P*-values for comparisons within subgroups are from Chi-Square Tests meeting PA. For SB, none of the comparison within subgroups were significant.

Overall, 62.2% of participants met PA recommendations (shown in Figure 1).

Men, participants in the younger age category (70–74 years), and those in the lower BMI category (<25 kg/m^2^) met PA recommendations more often (all *p* < 0.001 in univariate chi-square tests). Specifically, 71.6% of men met PA recommendations whereas only 56.4% of women did. The proportion of participants meeting PA recommendations decreased from 67.8% at ages 70–74 years to 54.7% for ages 75+. Of the participants with BMI ≥ 25 kg/m^2^, 58.0% reported to meet PA recommendations, while in the <25 kg/m^2^ category, 68.5% met PA.

With regard to country, prevalence of meeting PA recommendations was as follows: Austria 74.4% (148/199), France 51.0% (153/300), Germany 65.6% (229/349), Portugal 46.5% (140/301), and Switzerland 66.7% (671/1,006).

### Prevalence of SB

Overall, 37.1% of participants classified as being sedentary (spent ≥ 5.5 h/day with SB; shown in Figure 1).

There was no significant univariate differences due to age, sex and country with regard to SB with a consistent proportion of about one third of participants spending ≥5.5 h/day with SB.

### Prevalence for the Combination of Both Behavioral Patterns

**Figure 2:**
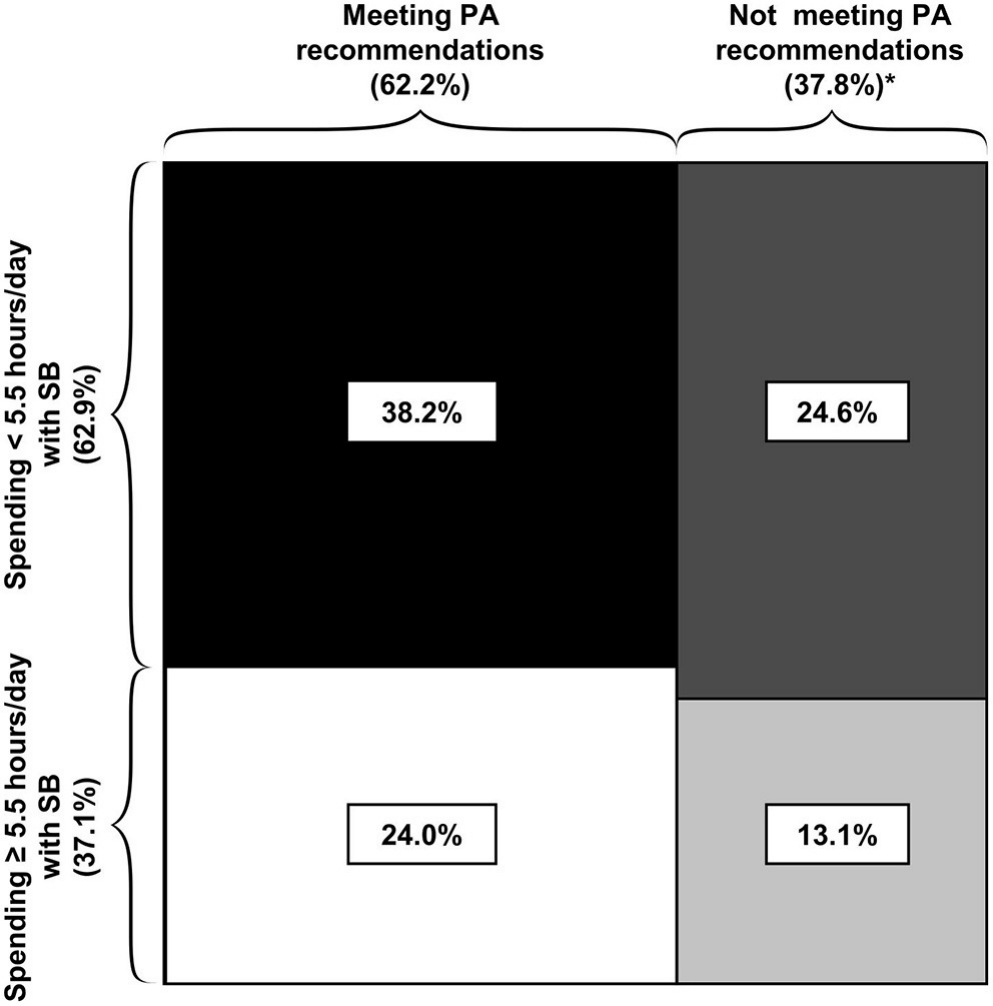
Prevalence within the four groups of combined physical activity and sedentary behavior patterns. *Differences in the sum are due to rounding. Areas reflect the number of participants in corresponding group.

We grouped participants into four categories based on combined patterns of PA and SB (shown in Figure 2): 24.0% met PA recommendations and at the same time classified as being sedentary (spent ≥5.5 h/day with SB); 38.2% met PA recommendations and at the same time spent <5.5 h/day with SB; 24.6% did not met PA recommendations and spent <5.5 h/day with SB; 13.1% did not meet PA recommendations and were sedentary.

For Portugal, the highest prevalence rate was in the category “not meeting PA recommendations/low SB”, while for all other countries the highest prevalence rate was in the “meeting PA recommendations/low SB” group (Supplement 4).

### Multivariable Logistic Regression Model for Odds of Meeting PA Recommendations

Participants had greater odds of meeting PA recommendations with each additional point on the SPPB score (OR= 1.23; 95%CI: 1.14, 1.34; Table 2).

**Table 2:** Multivariate logistic regression models, a) odds of meeting PA recommendations at baseline, b) odds of spending ≥5.5 hours/day with SB at baseline.

	**a) odds of meeting PA recommendations**			**b) odds to spend ≥5.5 hours/day with SB**		
	**(*n* = 2,111)**			**(*n* = 2,111)**		
	**OR**	**95% CI**	***P*** **-value**	**OR**	**95% CI**	***P*** **-value**
**Age (yrs)**	0.93	0.90, 0.95	<0.0001	1.01	0.98, 1.03	0.6409
**Female**	0.53	0.40, 0.71	<0.0001	0.87	0.66, 1.14	0.3029
**Prior Fall**	1.09	0.89, 1.32	0.4051	1.07	0.89, 1.29	0.4776
**BMI (kg/m^2^)**	0.93	0.91, 0.95	<0.0001	1.03	1.00, 1.05	0.0317
**Current smoker**	0.79	0.52, 1.19	0.2632	1.35	0.92, 1.96	0.1219
**Years of education (years)**	0.96	0.94, 0.99	0.0044	1.02	0.99, 1.04	0.1582
**MoCA score (continuous)**	0.93	0.90, 0.96	<0.0001	1.04	1.01, 1.08	0.0119
**Comorbidities (continuous)**	0.86	0.69, 1.05	0.1408	1.14	0.93, 1.39	0.2070
**Polypharmacy (≥5 medications)**	1.12	0.88, 1.43	0.3484	1.00	0.80, 1.27	0.9523
**Geriatric depression Scale score (GDS, continuous)**	0.91	0.87, 0.96	0.0001	1.03	0.99, 1.08	0.1424
**Living alone**	0.99	0.81, 1.22	0.9172	1.08	0.89, 1.31	0.4441
**SPPB score (continuous)**	1.23	1.14, 1.34	<0.0001	0.97	0.90, 1.05	0.4261
**Grip Strength dominant hand (continuous)**	1.00	1.00, 1.01	0.4365	1.00	1.00, 1.01	0.4851
**Country**						
Austria	1.17	0.81, 1.70	0.4079	1.00	0.72, 1.38	0.9893
France	0.74	0.54, 0.99	0.0459	1.04	0.78, 1.39	0.7912
Germany	0.83	0.62, 1.10	0.1955	0.88	0.67, 1.15	0.3456
Portugal	0.58	0.40, 0.84	0.0034	0.89	0.63, 1.26	0.5110
Switzerland	*Reference*			*Reference*		

*Prior fall: reporting of a fall in the 1-year period before study start. BMI: Body mass index, calculated as weight in kilograms divided by height in meters squared (kg/m^2^). BMI values ≥25 reflect overweight and values ≥30 obesity. MoCA: Montreal Cognitive Assessment, screening test for mild cognitive dysfunction with a range of 0 to 30 points, in which higher scores are better and scores >26 suggest normal cognitive function. MMSE: Mini-Mental State Examination, measures cognitive impairment with a range of 0 to 30 points, in which higher scores are better and scores >24 suggest normal cognitive function. MMSE of ≥24 was one of the inclusion criteria for the DO-HEALTH clinical trial. Multimorbidity was defined as ≥2 comorbidities. Comorbidities were assessed with a self-administered questionnaire to assess comorbidities (Sangha's Scores). GDS: Geriatric Depression scale, Questionnaire to assesses depression. Scores range from 0 to 15, 0–5 points are considered normal, 5–10 points translate to light to moderate depression, 11–15 to severe depression. SPPB: Short Physical Performance Battery, standardized assessment battery to test lower extremity function. Scores range from 0 to 12, in which higher scores are better. Grip Strength of the dominant hand was measured with a Martin Vigorimeter (in kilopascal, kPa). Multivariate logistic regression models: design variables of the DO-HEALTH study were age, sex, experience of a fall prior to inclusion, and country of residence. Additional covariates were: BMI, current smoking, having a dog, taking care of a person, living alone, years of education, being depressed (GDS score), cognitive function (MoCA score), multimorbidity (presence of ≥2 comorbidities), polypharmacy (taking ≥5 medications), and physical function (SPPB score, Grip Strength of the dominant hand)*.

Participants had lower odds of meeting PA recommendations if being female (OR = 0.53; 95%CI: 0.40, 0.71), for each additional year of age (OR = 0.93; 95%CI: 0.90, 0.95), for each 1 kg/m^2^ increase in BMI (OR = 0.93; 95%CI: 0.91, 0.95), for each additional year of education (OR = 0.96; 95%CI: 0.94, 0.99), for each additional point on the MoCA score (OR = 0.93; 95%CI: 0.99, 0.96), and for each additional point on the GDS score (OR = 0.91; 95%CI: 0.87, 0.96).

Regarding country of residence, participants residing in Portugal had lower odds of meeting PA recommendations compared to Switzerland (OR = 0.58; 95%CI: 0.40, 0.84). Additional analysis revealed that participants who spent ≥5.5 h/day with SB had greater odds of meeting PA recommendations compared to participants who spent <5.5 h/day with SB (OR = 1.33; 95%CI: 1.09, 1.63; Supplement 5).

### Multivariable Logistic Regression Model for Odds of Spending ≥5.5 h/day With SB

Participants had greater odds of spending ≥5.5 h/day with SB for each 1 kg/m^2^ increase in BMI (OR = 1.03; 95%CI: 1.00, 1.05; Table 2) and for each additional point in the MoCA score (OR = 1.04; 95%CI: 1.01, 1.08). Additional analysis revealed that participants who met PA recommendations had greater odds of spending ≥5.5 h/day with SB (OR = 1.33; 95%CI: 1.09, 1.62; Supplement 6). Variance inflation factors (VIFs) ranged between 1.0 and 2.2, indicating little evidence of multi-collinearity.

## Discussion

In this cross-sectional study of relatively healthy adults aged 70 years and older recruited from the community in 5 European countries, at baseline 62.2% reported to meet PA recommendations and 37.1% classified as sedentary (reported to spend ≥5.5 h/day of SB).

There was an overlap between these groups, with 24.0% of those meeting PA recommendations also reaching the threshold of being sedentary. Notably, 38.2% of those meeting PA recommendations also were below the sedentary threshold.

Meeting PA recommendations correlated positively with better physical function, and negatively with older age, being female, higher BMI, better education, better cognition, worse mental health, and residing in Portugal. Spending ≥5.5 h/day with SB was associated with higher BMI and better cognition.

Consistent with our findings, Bauman et al. (32) reported a lower prevalence of meeting PA with higher age within the World Health and SAGE Surveys: While less than a quarter of participants in the age group 60–69 years reported not meeting PA recommendations, this number rose to 30–40% among ages 70–79 years and to almost half of the population for ages 80+. Also, our findings are consistent with prior reports stating that men meet PA recommendations more often than women (33).

Regarding country-specific reports, our findings corroborate the SHARE study data on the variability of meeting PA recommendations between countries for older adults in Europe, which varied between 55 and 83% in SHARE (within 10 European countries at wave four, including cohort data of Switzerland, Austria, and France), and 46.5–74.4% in DO-HEALTH (8). Notably, compared to the European data, within three national surveys among older adults aged 65 and older residing in the USA (NHANES, BRFSS, and NHIS), prevalence of meeting PA recommendations has been reported to be lower: between 27 and 44% (34).

Our findings are also in line with prior studies suggesting that a higher BMI, decreased physical function, and lower mental health is associated with less engagement in PA (8, 35, 36).

In contrast to our findings, previous research reported a higher likelihood for meeting PA recommendations for participants with a higher education level (8). In DO-HEALTH, we found that for each additional year of education, the odds to meet PA recommendations decreased. This may in part be explained by the results of a systematic review among studies including cognitively healthy adults aged 60 years and older, which suggested that the association of better education with higher PA levels depends on the type of PA and may be more pronounced for PA behaviors that presuppose knowledge about associated health-benefits and accessibility (37).

Previously, exercise has been shown to improve cognitive function in healthy as well as in cognitively impaired older adults (38). In DO-HEALTH, better MoCA scores were associated with less favorable PA behaviors. As DO-HEALTH was not a population based study and had an inclusion criteria of MMSE ≥ 24, a cut-point generally considered to indicate normal cognitive function, our findings need to be interpreted with caution.

DO-HEALTH participants reported spending most PA time with light PA, such as walking—followed by gymnastics (yoga, stretching, figure training) and “other activities”. Notably, recent findings suggest that already engaging in light PA is reducing pre-mature mortality (3).

Pooling data from six countries, Harvey et al. (39) reported that on average 59% of older adults reported >4 h/day of SB, which is somewhat higher as reported in DO-HEALTH (37% overall) and likely reflective of the target population of relatively healthy adults age 70 and older in DO-HEALTH. However, similar to an European study investigating nationally representative samples aged 15 years and older, DO-HEALTH found that the prevalence rates of SB varied between European countries with high amounts of SB being more prevalent in Mediterranean countries than in more Northern countries (40).

Regarding correlates of SB, DO-HEALTH confirms prior studies among older adults that having a higher BMI is associated with greater levels of subjectively and objectively measured SB (35, 41).

In DO-HEALTH, we found that better cognition was associated with higher odds for spending ≥5.5 h/day with SB. The association of SB with cognitive function has not been studied extensively among healthy older adults and findings remain inconclusive as most studies did not adjust for PA (42). Further, the association of SB with cognitive function has been found to depend on the type of SB, e.g., whether the activity is passively watching TV, or to actively use a computer or reading (43). In DO-HEALTH, participants reported an overall median of 1.5 h/day of watching TV, which is less than half of the time that has previously been reported to be associated with tremendous health effects independently from PA in older adults (44).

Previous research including objective assessment of SB indicated that high amounts of SB may be associated with unfavorable health-related outcomes independent of engagement in PA (44). Thus, the assessment of both, PA and SB, appears to be relevant to health at older age.

The two latest population-based health surveys conducted in Switzerland considering PA and SB also reported similar prevalence around 50% of meeting PA recommendations and at the same time low SB behavior for adults aged 65–74 years as we found in DO-HEALTH (24).

For subgroups, we found that men more often met PA recommendations while at the same time reporting high amounts of SB, but women reported more often not meeting PA recommendations while spending <5.5 h/day with SB. This gender difference was also reported among a Dutch cohort (35). It has been speculated that this may be linked to traditional roles, such as women being less sedentary due to their household tasks and men having a more sedentary working history (18, 35).

Possibly reflecting such trade-offs in an overall very active older population, DO-HEALTH participants who met PA recommendations had a significantly greater odds to spend ≥5.5 h/day with SB.

Further research using longitudinal data is needed to clarify the impact of PA and PA intensity in relation to SB on health outcomes within this well characterized European population.

In DO-HEALTH, PA and SB was measured by self-report, but not by objective measures of PA such as accelerometer. Thus, we cannot exclude over- or underreporting of PA and SB. However, we used a highly validated assessment (NHS PAQ) (22), which was applied in a standardized way in all 5 recruitment countries. Another limitation is that participants were selected to be relatively healthy and active to be enrolled in DO-HEALTH. Therefore, they may not reflect the community-dwelling population aged 70 and older at a population-based level, and our findings need to be interpreted with caution.

Finally, as no established cut-off for SB exists (19), our findings related to SB need further validation.

In conclusion, the study population of DO-HEALTH represents a generally very active older adult population with a majority of participants meeting PA recommendations and spending <5.5 h/day with SB. However, PA profiles were less favorable in subgroups of older age, female sex and higher BMI. In addition, regarding the combined behavioral patterns of PA and SB, about half of the participants were either meeting PA recommendations or reporting <5.5 h/day of SB. Therefore, our findings support that individualized public health efforts may be warranted even in active older adults with similar characteristics as the DO-HEALTH participants.

## Data Availability Statement

The datasets presented in this article are not readily available because in a first step, no data will be made available to researchers external to DO-HEALTH Research Group to allow primary researchers to fully exploit the dataset. The data will be shared in a second step according to a controlled access system. Requests to access the datasets should be directed to HB-F, heike.bischoff@usz.ch.

## Ethics Statement

The studies involving human participants were reviewed and approved by Kantonale Ethikkommission Zurich, Switzerland. The patients/participants provided their written informed consent to participate in this study.

## Author Contributions

MM: substantial contribution to the design of the study, substantial contribution to the analysis and interpretation of data, writing of the manuscript, final approval of the version to be published, and agreement to be accountable for all aspects of the work in ensuring that questions related to the accuracy or integrity of any part of the work are appropriately investigated and resolved. UM: substantial contribution to the design of the study, the interpretation of data, critically revising of the manuscript, final approval of the version to be published, and agreement to be accountable for all aspects of the work in ensuring that questions related to the accuracy or integrity of any part of the work are appropriately investigated and resolved. WL and NM: substantial contribution to data analysis, critically revising the manuscript, final approval of the version to be published, and agreement to be accountable for all aspects of the work in ensuring that questions related to the accuracy or integrity of any part of the work are appropriately investigated and resolved. MG and RK: substantial contribution to the interpretation of the results, critically revising the manuscript, final approval of the version to be published, and agreement to be accountable for all aspects of the work in ensuring that questions related to the accuracy or integrity of any part of the work are appropriately investigated and resolved. RM and AE: substantial contribution to acquisition of data, critically revising the manuscript, final approval of the version to be published, and agreement to be accountable for all aspects of the work in ensuring that questions related to the accuracy or integrity of any part of the work are appropriately investigated and resolved. EO: substantial contribution to the interpretation of the results, advising expert for data analysis, critically revising the manuscript and presentation of data, final approval of the version to be published, and agreement to be accountable for all aspects of the work in ensuring that questions related to the accuracy or integrity of any part of the work are appropriately investigated and resolved. HB-F: PI of the DO-HEALTH clinical trial, PI and leading supervisor of the study, conception and design of the study and substantial contribution to the interpretation of the results, critically revising the manuscript, final approval of the version to be published, and agreement to be accountable for all aspects of the work in ensuring that questions related to the accuracy or integrity of any part of the work are appropriately investigated and resolved. All authors contributed to the article and approved the submitted version.

## Funding

The DO-HEALTH study was funded by the Seventh Research Framework Program of the European Commission (Grant Agreement No. 278588), and within this framework, also by the University of Zurich (Chair for Geriatric Medicine and Aging Research), DNP, Roche, NESTEC, Pfizer and Streuli. The funding/supporting organizations had no role in the design and conduct of the study, including collection, management, analysis, and interpretation of the data, as well as preparation, review, or approval of the manuscript, or decision to submit the manuscript for publication. The further use of DO-HEALTH data study reported in this manuscript was not funded.

## Conflict of Interest

The authors declare that the research was conducted in the absence of any commercial or financial relationships that could be construed as a potential conflict of interest.

## Publisher's Note

All claims expressed in this article are solely those of the authors and do not necessarily represent those of their affiliated organizations, or those of the publisher, the editors and the reviewers. Any product that may be evaluated in this article, or claim that may be made by its manufacturer, is not guaranteed or endorsed by the publisher.
