# Supplementary material for: Prevalence of Physical Activity and Sedentary Behavior Patterns in Generally Healthy European Adults Aged 70 Years and Older—Baseline Results From the DO-HEALTH Clinical Trial
Source: Front Public Health. 2022 Apr 14;10:810725. doi: 10.3389/fpubh.2022.810725 (PMC9046658; doi:10.3389/fpubh.2022.810725)
Supplement: Supplementary file 1 [file Data_Sheet_1.pdf]

Supplementary Material

1 NHS physical activity questionnaire (NHS PAQ)

Question 1:

q.1: "What is your usual walking pace outdoors?"

- ☐ slow, <3km/h
- ☐ medium, 3-4.5km/h
- ☐ brisk, 4.5-6km/h
- ☐ fast, >6km/h

➔ direct translation of the answers into a categorical variable with 4 response options

Question 2:

q.2: "How many flights of stairs (not individual steps) do you climb daily?"

- ☐ ≤ 2
- ☐ 3-4
- ☐ 5-9
- ☐ 10-14
- ☐ 15 or more

➔ direct translation of the answers into a categorical variable with 5 response options

Question 3:

q3.1 to q3.11: "During the past year, what was your average time per week spent at each of the following recreational activities?"

Supplementary Table 1. Wording of q3.1 to q3.11

Question no.	Activity
q3.1	Walking for exercise or walking to work
q3.2	Jogging (Slower than 6 min/km)
Q3.3.	Running (6 minutes/km or faster)
q3.4	Bicycling (include stationary machine)
q3.5	Tennis, squash, racquetball
q3.6	Lap swimming
q3.7	Other aerobic exercise (aerobic, dance, ski or stair machine, etc.)
q3.8	Lower intensity exercise (yoga, stretching, toning)
q3.9	Other vigorous activities (e.g., lawn mowing)
q3.10	Weight training or resistance exercises (free weights or machines) – arm weights
q3.11	Weight training or resistance exercises (free weights or machines) – leg weights

➔ **Answer categories:** ***average time/week:***

Supplementary Table 2. Coding of reported times

Category offered in questionnaire (per week)	Value used for coding of the sums of the activities	Comment / Calculation:
0 minutes	0 minutes	as reported
1-4 minutes	2.5 minutes	average of 1 and 4 minutes (5/2=2.5)
5-19 minutes	12 minutes	average of 5 and 19 minutes (24/2=12)
20-59 minutes	40 minutes	average of 20 and 59 minutes (79/2=39.5 → rounded to 40)
1 hour	60 minutes	as reported
1-1.5 hours	75 minutes	1 hour + average of 30 min (1hour + 15 min = 75 min)
2-3 hours	150 minutes	average of 2 and 3 hours (120+180=300; 300/2=150)
4-6 hours	300 minutes	average of 4 and 6 hours (240+360=600; 600/2=300)
7-10 hours	510 minutes	average of 7 and 10 hours (420+600=1020; 1200/2=510)
11+ hours	660 minutes	set at 11 hours = 660 minutes

<u>Question 4:</u>

q4.1 to q4.5: “On average, how many hours per day do you spend:”

Supplementary Table 3. Wording of q4.1 to q4.5

Question no.	Activity
q4.1	Standing or walking around at work
q4.2	Standing or walking around at home
q4.3.	Sitting at work or while driving*
Q4.4	Sitting at home while watching TV
Q4.5	Sitting at home (e.g., reading, while eating, sitting at desk)

*in a retired / non-working population, this question may relate to seated activities outside of home

➔ **Answer categories:** ***average time/day:***

Supplementary Table 4. Coding of reported times

Category offered in questionnaire (per day)	Value used for coding of the sums of the activities	Comment / Calculation:
0 minutes	0 minutes	as reported
1 hour	60 minutes	as reported
2-5 hours	210 minutes	average of 2 and 5 hours (120+300=420; 420/2=210)
6-10 hours	480 minutes	average of 6 and 10 hours (360+600=960; 960/2=480)
11-20 hours	930 minutes	average of 11 and 20 hours (660+1200=1860; 1860/2=930)

21-40 hours	1830 minutes	average of 21 and 40 hours (1260+2400=3660; 3660/2=1830)
41-60 hours	3030 minutes	average of 41 and 60 hours (2460+3600=6060; 6060/2=3030)
61-90 hours	4530 minutes	average of 61 and 90 hours (3660+5400=9060; 9060/2=4530)
90+ hours	5400 minutes	set at 90 hours = 5400 minutes

*Note: 1 day = 24 hours, 1 week = 168 hours

Question 5:

q5: "On average, on how many days per week do you usually exercise (incl. brisk walking or other vigorous activities)?"

- ☐ never
- ☐ 1 day
- ☐ 2 days
- ☐ 3 days
- ☐ 4 days
- ☐ 5 days
- ☐ 6 days
- ☐ 7 days

➔ **direct translation of the answers into a continuous variable**

2 Overview assignment of METs to tasks

- The values for the METs were taken from the Compendium of Physical Activities of 2011 (see supplementary table 7).(1)
- For q3.1, different METs were assigned in relation to the walking pace outdoors as reported in q1.
- For climbing stairs (q2) we assigned 8 METs and for the time to climb one flight of stairs we used 0.002 hours as proposed by Wolf et al. (1994).(2)

References:

(1) Ainsworth BE, Haskell WL, Herrmann SD, et al. 2011 Compendium of Physical Activities: a second update of codes and MET values. Medicine and science in sports and exercise. 2011;43(8):1575-1581

(2) Wolf AM, Hunter DJ, Colditz GA, et al. Reproducibility and validity of a self-administered physical activity questionnaire. International journal of epidemiology. 1994;23(5):991-999.

Supplementary Table 5. METs assigned to tasks from the NHS

NHS question	Activity as worded in the NHS	Classification of activity level	Compendium description	MET value assigned
**q3.1**	Walking or hiking outdoors including walking to work if walking speed = “easy/casual”	**Light PA**	Compendium 2011, code 17151: ”walking, less than 2.0 mph, level, strolling, very slow”; value supported by literature	**2.5**
**q3.1**	Walking or hiking outdoors including walking to work if walking speed = “normal/ average”	**Light PA**	Compendium 2011, code 17170: “walking, 2.5 mph, level, firm surface”; value supported by literature	**3.0**
**q3.1**	Walking or hiking outdoors including walking to work if walking speed = “brisk”	**Moderate PA**	Compendium 2011, code 17270: “walking, to work or class, Taylor Code 015”; value supported by literature	**4.0**
**q3.1**	Walking or hiking outdoors including walking to work if walking speed = “very brisk”	**Moderate PA**	Compendium 2011, code 17220: “walking, 4.0 mph, level, firm surface, very brisk pace”; value supported by literature	**5.0**
**q3.2**	Easy Jogging (less than 6.2mph, less than 10km/h)	**Vigorous PA**	Compendium 2011, code 12020 “jogging, general”; value estimated	**7.0**
**q3.3**	Running (6.2 mph or faster, 10km/h or faster)	**Vigorous PA**	Compendium 2011, code 12150: “running, Taylor code 200)”; value supported by literature	**8.0**
**q3.4**	Bicycling	**Moderate PA**	Compendium 2011, code 01010 “bicycling, <10 mph, leisure, to work or for pleasure, Taylor Code 115”; value supported by literature	**4.0**
**q3.5**	Tennis, squash, or racquetball	**Vigorous PA**	Compendium 2011, code 15530 “racquetball general, Taylor Code 470”; value supported by literature	**7.0**
**q3.6**	Lap swimming	**Moderate PA**	Compendium 2011, code 18240 “swimming laps, freestyle, front crawl, slow, light or moderate effort”; value supported by literature	**5.8**
**q3.7**	other aerobic exercise (e.g. aerobics/aerobic dance/rowing machine, stepper, cross-trainer	**Moderate PA**	Compendium 2011, code 03020, “aerobic dancing, low impact, moderate effort”; value supported by literature	**5.0**
**q3.8**	Gymnastics (e.g. yoga, stretching, figure training)	**Light PA**	Compendium 2011, code 2150, “Yoga, Hatha”; value supported by literature	**2.5**
**q3.9**	other activities (e.g. lawn moving)	**Moderate PA**	Compendium 2011, code 08095, “mowing lawn, general, value supported by literature	**5.5**
**q3.10**	Strength training arms	**Moderate PA**	Compendium 2011, code 02022, “calisthenics, e.g. pushups, sit ups, pull up, moderate effort”; value supported by literature	**3.8**
**q3.11**	Strength training legs	**Moderate PA**	Compendium 2011, code 02022, “calisthenics, e.g. pushups, sit ups, pull up, moderate effort”; value supported by literature	**3.8**
**q4.1**	Standing or walking around at work or away from home	**Light PA**	Compendium 2011, code 17161, “walking from house to car or bus, from car or bus to go places, from car or bus to and from the worksite”; value estimated	**2.5**
**q4.2**	Standing or walking around at home	**Light PA**	Compendium 2011, code 1750, “walking, less than 2.0 mph, level, strolling, very slow”; value supported by literature	**2.0**
**q4.3**	Sitting at work or while driving	**SED**	Compendium 2011, code 07022, “sitting quietly, fidgeting, general fidgeting hands”; value supported by literature	**1.5**
**q4.4**	Sitting while watching TV	**SED**	Compendium 2011, code 07020, “sit, watch television”; value supported by literature	**1.3**
**q4.5**	Sitting at home (e.g. reading, eating, at the desk)	**SED**	Compendium 2011, code 07022, “sitting quietly, fidgeting, general fidgeting hands”; value supported by literature	**1.5**

3 NHS PAQ responses

Supplementary Table 6. Responses of NHS PAQ, overall and stratified if meeting PA recommendations or not (a) and by spending ≥5.5 hours/day with SB or not (b) separately

NHS reporting behavior stratified by a) meeting PA recommendations in hours/week, and b) spending ≥5.5 hours/day with SB

		Overall	Table 2a meeting PA recommendations, hrs/week N=2155		Table 2b spending ≥5.5 hours/day with SB N=2155	
			not meeting PA recommendations	meeting PA recommendations	<5.5 hours/day	≥5.5 hours/day
**n (%)**		**2155**	814 (37.8)	1341 (62.2)	1355 (62.81)	800 (37.09)
**Active days/week,** number of days; median [IQR]		3 [1, 5]	1 [0, 3]	3 [2, 5]	3 [1, 4]	3 [1, 5]
**Usual walking pace outdoors,** n (%)*						
	slow, <3km/h	342 (15.9)	219 (26.9)	123 (9.2)	220 (16.2)	122 (15.3)
	medium, 3-4.5km/h	1175 (54.5)	534 (65.6)	641 (47.8)	735 (54.2)	440 (55.0)
	brisk, 4.5-6km/h	612 (28.4)	58 (7.1)	554 (41.3)	382 (28.2)	230 (28.8)
	fast, >6km/h	26 (1.2)	3 (0.4)	23 (1.7)	18 (1.3)	8 (1.0)
**Stairs flights climbed/day,** n (%)*						
	≤ 2	691 (32.01)	336 (41.3)	355 (26.5)	453 (33.4)	238 (29.8)
	3-4	517 (24.0)	195 (24.0)	322 (24.0)	323 (23.8)	194 (24.3)
	5-9	441 (20.5)	138 (17.0)	303 (22.6)	274 (20.2)	167 (20.9)
	10-14	257 (11.9)	79 (9.7)	178 (13.3)	158 (11.7)	99 (12.4)
	15 or more	249 (11.6)	66 (8.1)	183 (13.7)	147 (10.9)	102 (12.8)
**Leisure time PA, hours/week,** median [IQR]						
	Walking to work or for recreation/exercise	2.50 [1.00, 5.00]	1.25 [0.67, 5.00]	5.00 [1.25, 8.50]	2.50 [1.00, 5.00]	5.00 [1.25, 8.50]
	Jogging, <6km/h	0.00 [0.00, 0.20]	0.00 [0.00, 0.00]	0.00 [0.00, 0.00]	0.00 [0.00, 0.67]	0.00 [0.00, 0.01]
	Running, >6km/h	0.00 [0.00, 0.20]	0.00 [0.00, 0.00]	0.00 [0.00, 0.00]	0.00 [0.00, 0.67]	0.00 [0.00, 0.20]
	Cycling (outdoors or home trainer)	0.00 [0.00, 1.00]	0.00 [0.00, 0.20]	0.67 [0.00, 2.50]	0.00 [0.00, 1.00]	0.00 [0.00, 1.25]
	Tennis, Squash, Racquetball	0.00 [0.00, 0.00]	0.00 [0.00, 0.00]	0.00 [0.00, 0.00]	0.00 [0.00, 0.00]	0.00 [0.00, 0.00]

Lap swimming	0.00 [0.00, 0.04]	0.00 [0.00, 0.00]	0.00 [0.00,0.20]	0.00 [0.00, 0.4]	0.00 [0.00, 0.20]
Other aerobic exercise (e.g. aerobics, dance, cross-trainer, stepper)	0.00 [0.00, 0.04]	0.00 [0.00, 0.00]	0.00 [0.00, 0.67]	0.00 [0.00, 0.4]	0.00 [0.00, 0.00]
Gymnastics (Yoga, Stretching, figure training)	0.20 [0.00, 1.00]	0.00 [0.00, 0.67]	0.67 [0.00, 1.25]	0.20 [0.00, 1.00]	0.20 [0.00, 1.00]
Other activities e.g. lawn mowing	0.20 [0.00, 1.00]	0.00 [0.00, 0.20]	0.67 [0.00, 2.50]	0.20 [0.00, 1.00]	0.20 [0.00, 1.25]
Strength training arms	0.00 [0.00, 0.20]	0.00 [0.00, 0.00]	0.00 [0.00, 0.67]	0.00 [0.00, 0.20]	0.00 [0.00, 0.20]
Strength training legs	0.00 [0.00, 0.20]	0.00 [0.00, 0.00]	0.00 [0.00, 0.20]	0.00 [0.00, 0.20]	0.00 [0.00, 0.42]
**Standing or walking, hours/day,** median [IQR]					
at work/outside home	8.00 [3.50, 15.50]	3.50 [3.50, 8.00]	8.00 [3.50, 15.50]	3.50 [3.50, 8.00]	8.00 [3.50, 30.50]
at home	8.00 [3.50, 15.50]	8.00 [3.50, 15.50]	8.00 [3.50, 15.50]	3.50 [3.50, 8.00]	15.50 [8.00, 30.50]
**SB, hours/day,** median [IQR]					
at work/outside home/while driving	0.500 [0.50, 2.21]	0.50 [0.50, 1.14]	1.14 [0.50, 2.21]	0.50 [0.50, 1.14]	2.21 [1.14, 4.36]
seating at home	1.14 [0.50, 2.21]	1.14 [0.50, 2.21]	1.14 [0.50, 2.21]	1.42 [0.50, 1.14]	4.36 [2.21, 4.36]
seating while watching TV	1.14 [0.50, 2.21]	1.14 [0.50, 2.21]	1.14 [0.50, 2.21]	0.50 [0.50, 1.14]	2.21 [1.14, 4.36]
**total time spent with SB, hours/day,** median [IQR]	3.86 [2.14, 6.71]	3.43 [1.50, 6.64]	4.43 [2.14, 7.07]	2.43 [1.50, 3.86]	7.71 [6.64, 9.93]
**total time spent with leisure time PA, hours/week,** median [IQR]	7.5 [3.5, 13.17]	2.75 [1.28, 5.40]	11.00 [6.85, 16.87]	6.50 [3.17, 12.17]	8.55 [4.75, 14.18]
**total time spent with light PA, hours/week,** median [IQR]	18.5 [9.5, 38.7]	14.67 [8.00, 31.00]	21.00 [11.50, 43.50]	13.25 [8.00, 24.50]	33.50 [17.25, 60.75]
**total time spent with moderate PA, hours/week,** median [IQR]	2.56 [0.67, 7.33]	0.40 [0.00, 0.17]	5.67 [2.98, 10.03]	2.50 [0.67, 6.37]	3.19 [0.67, 8.50]
**total time spent with vigorous PA, hours/week,** median [IQR]	0.17 [0.5, 1.38]	0.06 [0.01, 0.03]	0.72 [0.10, 2.67]	0.21 [0.05, 1.42]	0.17 [0.05, 1.28]
**total time spent with moderate+vigorous PA, hours/week,** median [IQR]	3.80 [1.10, 9.60]	0.72 [0.17, 0.19]	7.68 [4.52, 12.72]	3.46 [1.16, 8.76]	5.05 [1.01, 10.77]

% are summed up in the columns

4 Prevalence of the four combinations of Physical Activity and Sedentary Behavior, in % per Subgroup

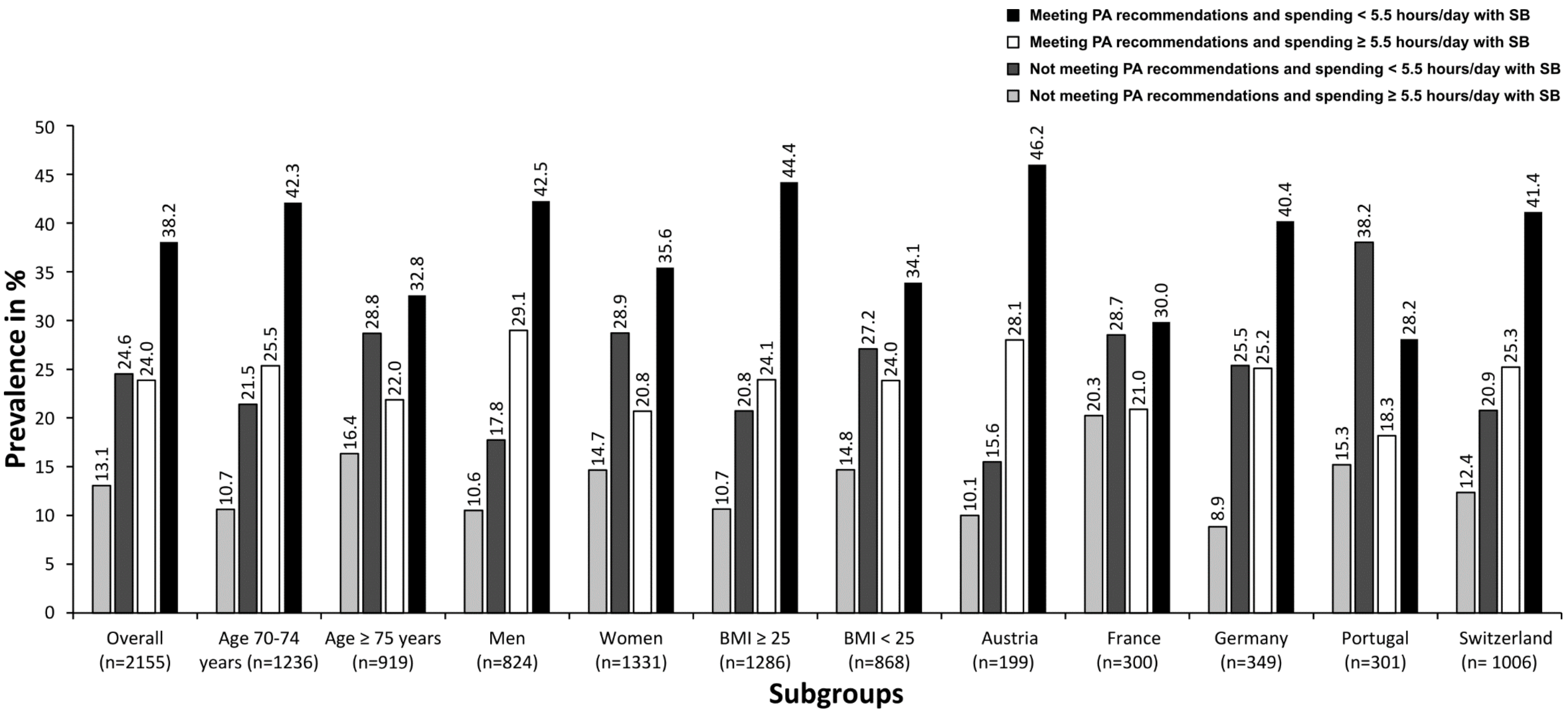

Supplementary Figure 1. Prevalence of the four combinations of Physical Activity and Sedentary Behavior, in % per Subgroup

5 Sensitivity analysis adjusting the PA model for SB

Supplementary Table 7. Multivariate logistic regression model: odds of meeting PA recommendations adjusted for SB

	odds of meeting PA recommendations n=2116		
	OR	95% CI	*P-value*
**Age [yrs]**	0.93	0.90, 0.95	<.0001
**Female**	0.54	0.40, 0.72	<.0001
**Prior Fall**	1.08	0.89, 1.31	0.4292
**BMI [kg/m2]**	0.93	0.91, 0.95	<.0001
**Current smoker**	0.78	0.51, 1.17	0.2256
**Years of education [years]**	0.96	0.94, 0.99	0.0036
**MoCA score (continuous)**	0.93	0.89, 0.96	<.0001
**Comorbidities (Shanga's score, continuous)**	0.85	0.69, 1.04	0.1155
**Polypharmacy (≥5 medications)**	1.124	0.88, 1.43	0.3451
**Geriatric Depression Scale score (GDS, continuous)**	0.91	0.87, 0.95	<.0001
**Living alone**	0.98	0.80, 1.21	0.8648
**SPPB score (continuous)**	1.24	1.14, 1.34	<.0001
**Grip strength dominant hand**	1.00	1.00, 1.01	0.4656
**Country**			
Austria	1.17	0.80, 1.69	0.0284
France	0.73	0.54, 0.99	0.2676
Germany	0.84	0.63, 1.11	0.9659
Portugal	0.59	0.41, 0.84	0.0121
Switzerland		*Reference*	
**spending ≥ 5.5 hours/day with SB**	1.33	1.09, 1.63	0.0049

6 Supplement 6: Sensitivity analysis adjusting the SB model for PA

Supplementary Table 8. Multivariate logistic regression model: odds of spending ≥ 5.5 hours/day with SB adjusted for PA

	**odds to spend ≥ 5.5 hours/day with SB** n=2122		
	OR	95% CI	*p-value*
**Age [yrs]**	1.01	0.99, 1.03	0.3955
**Female**	0.90	0.69, 1.18	0.4418
**Prior Fall**	1.07	0.89, 1.28	0.5073
**BMI [kg/m2]**	1.03	1.01, 1.05	0.0126
**Current smoker**	1.37	0.94, 1.99	0.1064
**Years of education [years]**	1.02	1.00, 1.05	0.1128
**MoCA score (continuous)**	1.05	1.01, 1.09	0.0059
**Comorbidities (Shanga's score, continuous)**	1.15	0.94, 1.40	0.1772
**Polypharmacy (≥5 medications)**	1.00	0.80, 1.26	0.9946
**Geriatric Depression Scale score (GDS, continuous)**	1.04	0.99, 1.09	0.0887
**Living alone**	1.08	0.89, 1.32	0.4341
**SPPB score (continuous)**	0.96	0.89, 1.03	0.2603
**Grip Strength dominant hand (continuous)**	1.00	1.00, 1.01	0.5140
**Country**			
Austria	0.99	0.72, 1.37	0.8653
France	1.06	0.79, 1.42	0.4412
Germany	0.89	0.67, 1.16	0.4317
Portugal	0.92	0.65, 1.30	0.6917
Switzerland		*Reference*	
**meeting PA recommendations**	1.33	1.09, 1.62	0.0053
